# 

**Published:** 1881-02-20

**Authors:** 


Entered at the Post-Office at Atlanta as second-class matter.

VOL. XI. FEBRUARY 20, 1881. NO. 2.

### THE SOUTHERN MEDICAL RECORD:

### A MONTHLY JOURNAL OF PRACTICAL MEDICINE.

Terms: $2 00 per Annum, in Advance; 4 Copies $6.00; Single Copies 20 Cents.

*"QUICQUID PRÆCIPIES ESTO BREVIS."*

